# Study of Paclitaxel-Treated HeLa Cells by Differential Electrical Impedance Flow Cytometry

**DOI:** 10.3390/bios4030257

**Published:** 2014-08-13

**Authors:** Julie Kirkegaard, Casper Hyttel Clausen, Romen Rodriguez-Trujillo, Winnie Edith Svendsen

**Affiliations:** 1 Department of Micro- and Nanotechnology, Technical University of Denmark, Oersteds Plads 345 East, DK-2800 Kgs. Lyngby, Denmark; E-Mails: julki@nanotech.dtu.dk (J.K.); romenrt@yahoo.es (R.R.-T.); Winnie.Svendsen@nanotech.dtu.dk (W.E.S.); 2 DELTA Dansk Elektronik, Lys & Akustik, Venlighedsvej 4, DK-2970 Hørsholm, Denmark

**Keywords:** electrical impedance spectroscopy, HeLa cells, paclitaxel, cell properties

## Abstract

This work describes the electrical investigation of paclitaxel-treated HeLa cells using a custom-made microfluidic biosensor for whole cell analysis in continuous flow. We apply the method of differential electrical impedance spectroscopy to treated HeLa cells in order to elucidate the changes in electrical properties compared with non-treated cells. We found that our microfluidic system was able to distinguish between treated and non-treated cells. Furthermore, we utilize a model for electrical impedance spectroscopy in order to perform a theoretical study to clarify our results. This study focuses on investigating the changes in the electrical properties of the cell membrane caused by the effect of paclitaxel. We observe good agreement between the model and the obtained results. This establishes the proof-of-concept for the application in cell drug therapy.

## 1. Introduction

Investigating cell responses to different drug therapies is a method used to determine the effectiveness of a drug candidate or to understand the resistive behavior of the cells towards drug treatments. Developing technologies within these fields can be used in areas, such as personalized medicine, where it is of high importance to monitor the effect of the therapeutic cancer drug on cancer cells. Several methods, which investigate cell response to drug treatment, are currently in force and typically assess whether a cell membrane has been breached. They mostly rely on vital stains, such as trypan blue, FITC Annexin V or propidium iodide [1]. The stained cells can be analyzed by microscopy or more advanced methods, like fluorescence-activated cell sorting (FACS) analysis. Other methods test the integrity of the cell by detecting the released intracellular material [2]. The various investigation methods usually probe the change in different properties of the cells due to the drug. A number of tools for measuring changes in membrane composition exist, e.g., fluorescence imaging of protein content [3], membrane potential and flow cytometry [4].

Characterizing cells by detecting dielectric changes by electrical impedance is a technique that has been investigated through several approaches, such as the Coulter counter [5] or impedance spectroscopy [6]. Lately, single-cell electrical impedance spectroscopy (EIS) [7,8,9] has been used to investigate cell suspensions. Single-cell EIS is a microfluidic technique used to probe electrical properties at the single cell level. In this technique, a microfluidic channel is used to handle the sample and direct it towards a set of electrodes where an AC electrical field, at multiple frequencies, is applied to the electrodes [9]. The method probes conformal changes (size, composition) of the sample in suspension, and it is able to characterize the biological sample properties in a label-free manner. Depending on the applied AC frequency, different properties of the sample can be probed, e.g., the cell size at low frequencies. A further development of the technology has led to the ability of applying a mixed signal of different frequencies.

The possibility of having EIS integrated into microfluidic systems is a relatively new approach, which has been demonstrated to have various applications within the characterization of biological samples [7,8,10,11]. The advantages of this integration are the better control of, e.g., the detection volume and electrode area, as well as the higher sensitivity of the system due to the same size range of the channel and particle. The technique has been widely used; from differentiation between red blood cells extracted from fish and human leukocytes [10] to the detection of DNA in droplets [12]. Gawad *et al.* [7] presented the first single-cell EIS microfluidic analysis system with a differential impedance detection scheme. They reported how erythrocytes and erythrocyte ghost cells could be differentiated with EIS, as well as particle size separation in continuous flow. The versatility of the differential EIS technology has been explored in a number of reports; it has been used to distinguish different yeast cells [13] and human blood cells of different kinds [8,14,15,16]. EIS has also been used to measure the effect of electrical lysis on yeast cells [17]. Furthermore, extensive modeling has been performed in order to elucidate how different sample properties influence the recorded impedance [18,19].

The cells used in this work are human cervical cancer cells of the well-known and documented HeLa cell line. EIS has previously also been used to investigate the electrical response from single stationary HeLa cells [20]. Paclitaxel is a cancer therapeutic drug, which induces cellular apoptosis of treated HeLa cells by stabilizing the microtubules of the cell, thus inhibiting cellular mitosis [21]. Kim *et al.* [21] investigated the size and morphological membrane changes of HeLa cells treated with paclitaxel by atomic force microscopy. The morphologic changes were reported to be torn and holed cell membranes with increased cellular surface roughness. As the membrane is perforated, extracellular fluid is allowed to enter the cell. When structural changes occur in the membrane, the electrical properties of the membrane will change consequently. Furthermore, Kim *et al.* [21] reported no significant changes in cell size due to treatment.

In this paper, the proof of concept of applying EIS as a method for cell differentiation between chemotherapeutic drug-treated and non-treated cells is shown using a custom-made microfluidic whole cell biosensor. This biosensor system has integrated coplanar microelectrodes with an optimized channel design for more accurate measurements while keeping the chip fabrication simple. A multi-channel lock-in amplifier is used to record a multi-frequency AC impedance signal of treated and/or non-treated HeLa cells suspended in phosphate buffered saline (PBS). The cells are mixed with polystyrene beads with a diameter of 4.5 µm. These beads act as a calibrating standard, and due to the small bead diameter relative to the HeLa cells, they do not influence the cell measurements. Furthermore, staining of the cells was performed to verify the reported drug effect on the cells. A theoretical model is used, together with the measurements, to interpret and validate the results. We demonstrate that EIS can be used to distinguish between the paclitaxel-treated and non-treated cells.

## 2. Experimental Section

### 2.1. Detection and Chip Design

**Figure 1:**
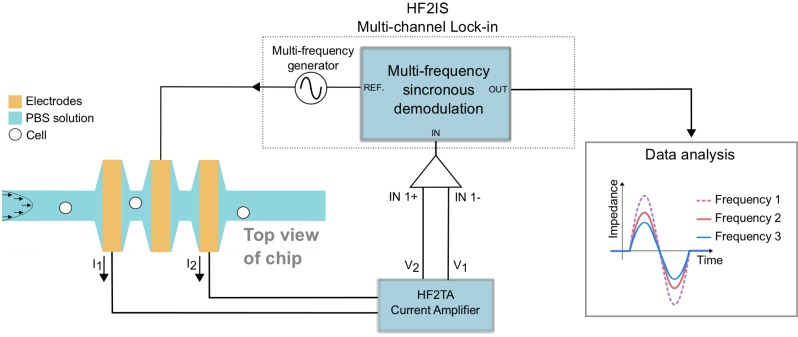
Schematic drawing of the setup. A multi-frequency lock-in amplifier is used to generate a signal and to detect the impedance. The signal generated between the electrodes in the channel is passed through a current trans-amplifier before it is returned to the lock-in amplifier. The detection unit consists of a microfluidic channel with expansions around the electrodes in order to decrease the overall impedance of the system, thus improving signal quality. The sample is pumped through the channel, and cells perturb the electrical impedance as they pass over the electrodes. Differential impedance between the two measuring electrodes is recorded for further analysis. The figure is not drawn to scale.

The system used in this work consists of an electrical detection scheme. It uses a differential electrode design, which consists of three coplanar electrodes on the bottom of a single microfluidic channel, as described by Gawad *et al.* [7]. Our design includes a channel expansion around the electrodes to obtain a larger electrode surface area and to reduce the overall impedance of the system (Figure 1). The channel is 30 µm wide and 30 µm high, and the expansion section around the electrodes is 15 by 50 µm, with the electrodes exposed in the channel. The electrode is 10 µm wide and 50 µm long. The larger electrode area also allows for larger potentials to be applied without risking cell lysis; therefore, a higher signal-to-noise ratio is obtained. The current density is focused between the electrodes, due to the narrow regions, thus ensuring the highest impedance response when the cell is halfway between two electrodes. No flow disturbances are observed, since the flow is laminar and the flow in the expansion sections is relatively low compared to that in the main channel.

### 2.2. HeLa Cell Culturing and Treatment

Human cervical cancer cells of the HeLa cell line (obtained from ATCC CCL-2, LGC Standards AB, Sweden) were cultured in a mixture of Eagle’s minimum essential medium (Sigma-Aldrich, Denmark) with 10% fetal bovine serum in an incubator with humidified air (5% CO_2_ at 37 °C) (Heracell 150, Thermo Scientific). Media was changed every 2 to 3 days, and cells were split once a week or when reaching 80% confluence.

The therapeutic cancer drug, paclitaxel (Sigma-Aldrich, Denmark), was used to treat and kill the HeLa cells. A drug concentration of 100 nM was used, and cells were treated over a period of 72 h to ensure sufficient time for the drug to react with the cells. This concentration was chosen based on previous studies, which showed that the majority of the cells were killed after exposure to paclitaxel for 72 h [22].

Cells were released from the culture flask with trypsin before the experiments, since the HeLa cells are an adherent cell line. Both cell cultures, treated and non-treated, were trypsinized (trypsin EDTA, Sigma-Aldrich, Denmark). The culture medium was exchanged with isotonic 1× phosphate buffered saline (PBS) (Sigma-Aldrich, Denmark) to a concentration of 0.5 × 10^6^ cells/mL.

### 2.3. Staining of Cells

Cells were stained with the vital cell stain, trypan blue (Sigma-Aldrich, Denmark), in order to visualize the viability of the cells. Dead cells are stained blue due to the perforated cell membrane, which allows the trypan blue to enter into the cell. Vital cells are not stained, since they have an intact membrane that inhibits the entrance of trypan blue. Cells suspended in PBS were stained with 10% v/v trypan blue for 5 min and analyzed using an inverted microscope using a CCD camera (Nikon Eclipse Ti, digital sight ds-2mbwc).

### 2.4. Chip Fabrication and Measurement Setup

One hundred-nanometer gold electrodes, with a 20-nm titanium layer for adhesion, were defined on 4-inch Pyrex wafers by a negative photolithography process (resist AZ5214e). On top of the electrodes, the channels were formed in SU-8 2075 (MicroChem, Germany) by photolithography [23,24]. For the experiments, the chips were reversibly sealed with a polydimethylsiloxane (PDMS) lid (Dow Corning, Germany), which was clamped to the chip by a customized holder. The holder also contained shielded connectors for the electrical readout. Grounding in the near proximity of the electrodes and coaxial cables with SMA connectors helped reduce the electrical noise.

### 2.5. EIS Measurements

Differential EIS measurements were performed by applying AC signals with amplitudes of 3 V (V_peak_) onto the center micro-electrode in the microfluidic channel. The impedance response was measured on the two outer electrodes. The impedance was recorded as a differential signal between the two outer electrodes. The current signal of the particle transitions was amplified by an HF2TA current amplifier (Zurich Instruments, Switzerland) and converted into an impedance signal and then detected by an HF2IS Impedance Spectroscope (Zurich Instruments, Switzerland). A computer recorded the detected impedance. The concepts of the setup and measured response are illustrated in Figure 1.

**Figure A1:**
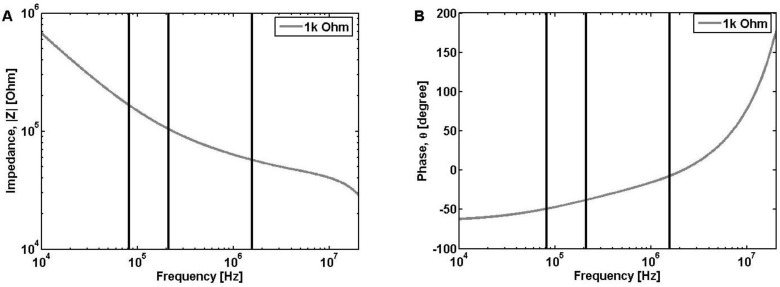
Bode plot. Frequency sweep of an electrode set from one of the chips used in this study. The black vertical lines represent the values of the frequencies used in the study, 82 kHz, 210 kHz and 1.57 MHz.

Prior to the experiments, the chip was filled with PBS. Frequency sweeps over the analyte between the electrodes were carried out in order to characterize the impedance response. The impedance is measured as differentiated in these experiments. Therefore, the frequency range of interest was selected where the derivative of the Bode plot was close to zero. The contribution from the resistance of the analyte is dominating in this region. Particles passing in the analyte over the electrodes will cause maximum impedance change [19] (see Appendix for Bode plots, Figure A1). Furthermore, the specific frequencies were selected in order to avoid contributions and unwanted interference from the harmonics.

To test the size resolution of the device samples, both 4.5- and 10-µm polystyrene beads (Polysciences, Inc., USA) were loaded into the chip. A concentration of 0.5 × 10^6^ beads/mL was used, and the sample was loaded with a Nexus 3000 syringe pump (Chemyx Inc., USA) at a rate of 0.4 µL/min. The measurements were carried out with three different frequencies simultaneously (82 kHz, 210 kHz and 1.57 MHz). The sample flow was verified by inverted optical microscopy. The analysis of the size resolution was followed by a viability study of the HeLa cells. Cells (both treated and non-treated) suspended in PBS at a concentration of 0.5 × 10^6^ cells/mL were mixed with 4.5-µm beads of the same concentration. The acquired data was analyzed using a custom-written script in MATLAB (The MathWorks Inc., USA).

### 2.6. Theoretical Calculations

**Figure 2:**
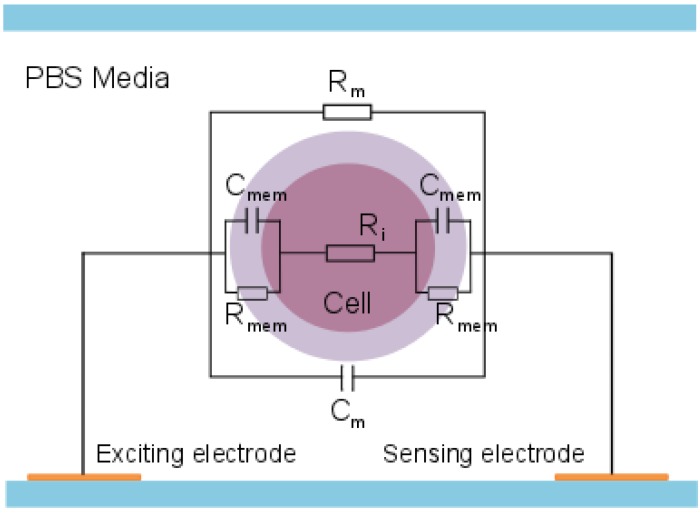
Schematic drawing of the equivalent circuit model.

The change in dielectric properties in the channel, caused by particles in the suspension passing over the electrodes, will give rise to a change in the measured impedance. Several models can be used to describe the electrical response of the system. The model used in this study is based on the Maxwell mixture theory (MMT) and is used to describe the behavior of the recorded impedance [18,19,25]. The model does not take the double-layer capacitance into account, as its influence is primarily in the very low frequency range [19]. The cell constant of the dual coplanar electrode layout was determined using conformal mapping (a three-step transformation), as described by Linderholm and Renaud [26] and Demierre *et al.* [23]. This is used in the calculations of the total impedance response (see the Appendix). The total impedance is given as 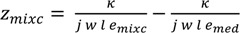, where *j*^2^ *=* −1, *l* is the length of the electrodes, *w* is the width of the electrodes, *k* is the cell constant of the electrode layout, *e_mixc_* is the complex permittivity of a cell in the volume between a pair of electrodes and *e_med_* is the complex permittivity of the liquid. An equivalent circuit model can be used as a complementary model to the MMT model [19]. Figure 2 illustrates the equivalent circuit model, where *R_m_* is the resistance of the medium, *R_i_* is the resistance of the cytoplasm, *R_mem_* is the resistance of the membrane, *C_mem_* is the capacitance of the membrane and *C_m_* is the capacitance of the medium.

**Figure 3:**
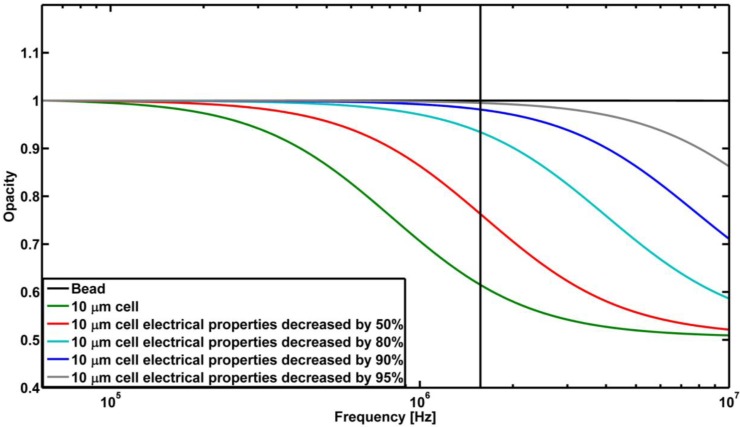
Calculated opacity response of a bead, an intact cell and cells with varying electrical properties of the membrane based on the MMT model. The values changed are 1/*σ_mem_* and ε*_mem_*. The vertical line represents the high frequency used in this work to investigate the paclitaxel-treated and non-treated HeLa cells. The low frequency used is 82 kHz.

Paclitaxel is known to stabilize the cytoskeleton, as well as to perforate the membrane. Furthermore, it can be assumed that treated and non-treated cells will have roughly the same size distribution [21]. The model can thus be used to describe the effect of paclitaxel by decreasing the membrane resistance and capacitance. We model the effect of membrane perforation as a decrease in the resistivity and permittivity of the membrane, assuming that the rest of the cell structure is intact. This corresponds to a decrease in the resistance and capacitance of the cell membrane in the equivalent circuit model. The used assumption is based on the reported effect of paclitaxel, *i.e*., an increase in surface roughness and membrane perforation. The modeling of the opacity as a function of frequency at varying electrical properties of the membrane is shown in Figure 3. The opacity is the ratio between the absolute impedance measured at the high frequency |*Z_high_*| and low frequency |*Z_low_*| of the passing particle, given as |*Z_high_*|/|*Z_low_*|. This parameter is known to reflect changes in the membrane and to reduce contributions from the size and vertical position of the particle in the channel [7,8]. Figure 3 implies that the impedance response of perforated cells is less dependent on the applied frequency.

The calculations were performed with the following parameters: ε_0_ = 8.854 × 10^−12^ F·m^−1^, *r_bead_* = 2.5 µm, **σ***_bead_* = 1 × 10^−3^ S·m^−1^, ε*_bead_* = 2.6ε_0_, *r_cell_* = 7.5 µm, *d_cell_* = 5 nm, **σ***_mem_* = 10 × 10^−8^ S·m^−1^, ε*_mem_* = 8.3ε_0_, **σ***_m_* = 1.6 S·m^−1^, ε*_m_* = 80ε_0_, **σ***_i_* = 0.6 S·m^−1^, ε*_i_* = 60ε_0_; where ε_0_ is the vacuum permittivity, *r* is the radius, *d* is the membrane thickness, **σ** is conductivity and ε is the permittivity. The subscripts: *bead*, *cell*, *mem*, *m* and *i*, signify the bead, cell, membrane, medium and cytoplasm of the cell, respectively.

## 3. Results and Discussion

### 3.1. System Resolution and Bead Separation

System characterization with beads of two different sizes was done in order to analyze the system resolution in terms of microscale size separation. Furthermore, the impedance signal from the smaller beads can be used as a calibrating standard to distinguish cell debris from cells when measuring on samples with whole cells.

The size resolution of the system was tested on a sample with polystyrene beads of 4.5 and 10 µm in diameter. The beads were suspended in a PBS solution at a concentration of 0.5 × 10^6^ beads/mL. The 10-µm beads were chosen, since their diameter is close to the diameter of HeLa cells (approximately 15 µm), but not large enough to clog the channel. It was observed that the bead diameter should be no larger than 1/3 of the channel width or height to avoid clogging. The 4.5 µm beads were chosen as the second bead size, because of the relatively low size compared to HeLa cells.

**Figure 4:**
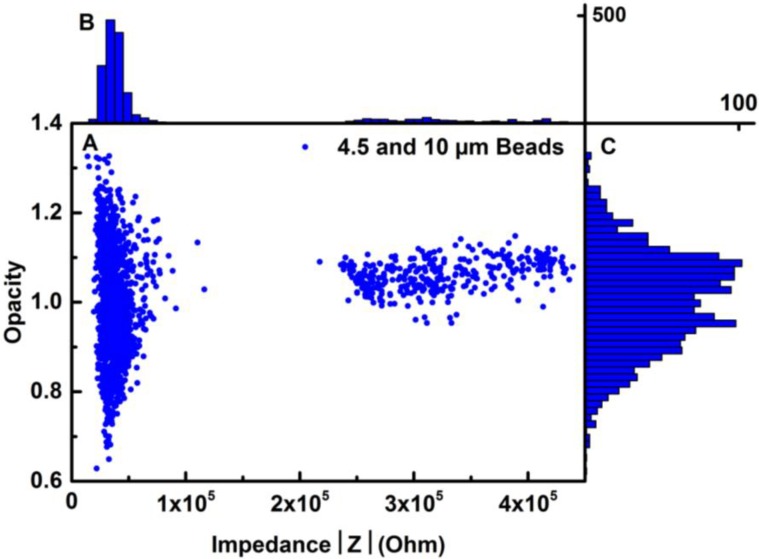
Data analysis of the impedance response of 4.5- and 10-µm beads. The measurements were normalized to one for comparison; the frequencies used were high frequency (HF) at 1.57 MHz and low frequency (LF) at 82 kHz. (**A**) Maximum differential impedance scatter plot of the opacity response as a function of the LF impedance. (**B**) Histogram distribution of the LF impedance. (**C**) Histogram distribution of the opacity.

Figure 4A is a maximum differential impedance scatter plot of the opacity as a function of the low frequency (LF) impedance signal. The figure shows two clearly separated populations that represent the 4.5- and 10-µm beads. The frequencies used are 1.57 MHz and 82 kHz. Figure 4B shows a histogram of the opacity of the beads normalized to one. In Figure 4C, a histogram of the LF impedance signal is plotted. The clearly-defined distribution around 0.4e5 Ω is the impedance from the 4.5-µm beads, while the distribution with a larger spread around 3.3e5 Ω corresponds to the impedance from the 10-µm beads. This was confirmed by optical microscopy of the electrode area during experiments.

The low frequency response reflects the size of the particles and, hence, any variation in the size. This explains the large distribution in the LF range for the 10-µm beads, since the beads have a coefficient of variance (COV) of 10%. This means that the 10-µm beads have a standard variation of ±1 µm. The 4.5-µm beads have a COV of 7%. Since the volume of a sphere is dependent on the radius to the power of three, the volume variation for the 10-µm beads has a larger impact on the measured impedance. The large spread of the 10-µm beads is reduced when looking at the opacity, since the impedance contribution of vertical displacement is reduced. On the other hand, the impedance distribution of the 4.5-µm beads is larger when looking at the opacity. This is probably due to their smaller size, thus a larger distribution in the detection volume. Although the beads were mixed in the same concentration, it is seen from Figure 4A, C that the count of the 4.5-µm beads is higher than that of the 10-µm beads. The smaller beads are more likely to enter the microfluidic channel, since they are less affected by obstructions in the inlet-channel interface and will sediment less. During the measurements, it was observed that large beads had a tendency to sediment in the inlet area and to get caught under the PDMS lid. These observations correspond to the measured data. The measurements showed that the system is capable of clearly distinguishing between 4.5- and 10-µm beads, and we can even detect the volumetric variation of the 10-µm beads. Based on these results, the 4.5-µm beads are suitable as a calibrating standard for the cell analysis. Furthermore, the maximum differential impedance of the 4.5-µm beads can be used as a cut-off for cell debris and electrical noise indicated by the clear separation from the larger beads.

### 3.2. Impedance Response of Paclitaxel-Treated Cells

HeLa cells were used as the cellular model for this experiment. The cells were chosen because of their relative easy culturing methods and stability. The chemotherapeutic drug, paclitaxel, was used as the chemotoxic agent, since its effect on HeLa cells is well documented. Differential EIS was used to test the viability of the cells.

**Figure 5:**
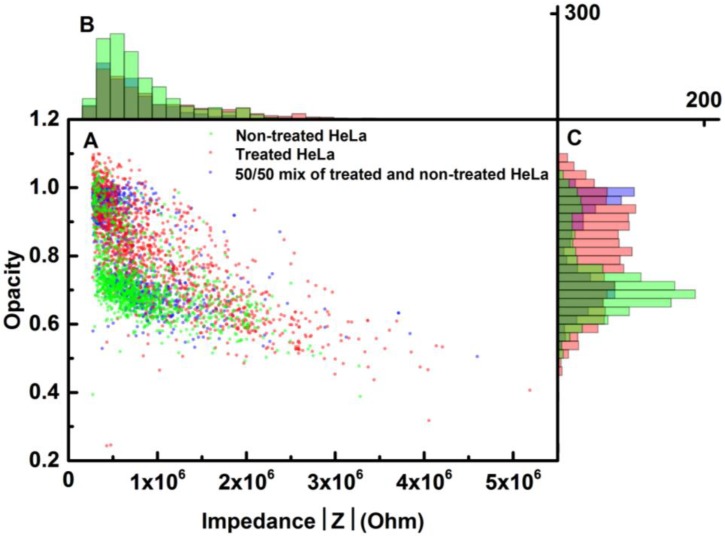
Data analysis of the impedance response of treated and non-treated cells; the frequencies used were HF at 1.57 MHz and LF at 82 kHz. (**A**) Maximum differential impedance scatter plot of the opacity response as a function of the LF impedance signal of the three different samples, where the bead data has been removed (see the Appendix). (**B**) Histogram distribution of the LF impedance signal. (**C**) Histogram distribution of the opacity of the different samples; beads and treated cells, beads and non-treated cells and beads plus treated and non-treated cells.

**Figure 6:**
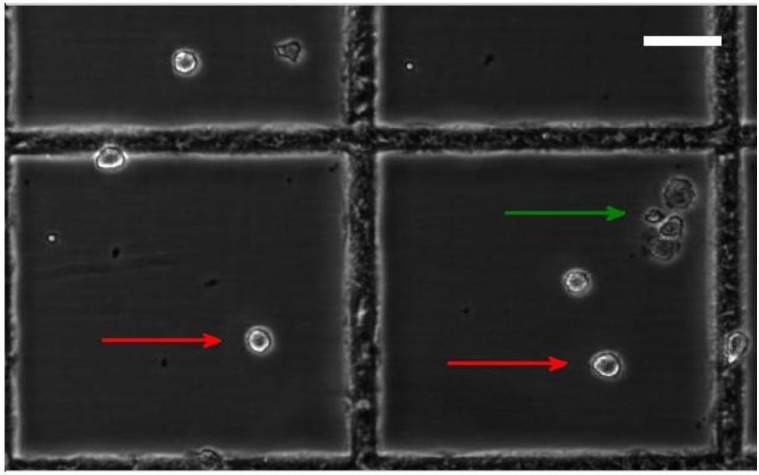
Optical image of the used cell sample with a 50/50 mix of treated and non-treated cells stained with trypan blue (scale bar: 50 µm). The red and green arrows are alive and dead cells.

**Figure A2:**
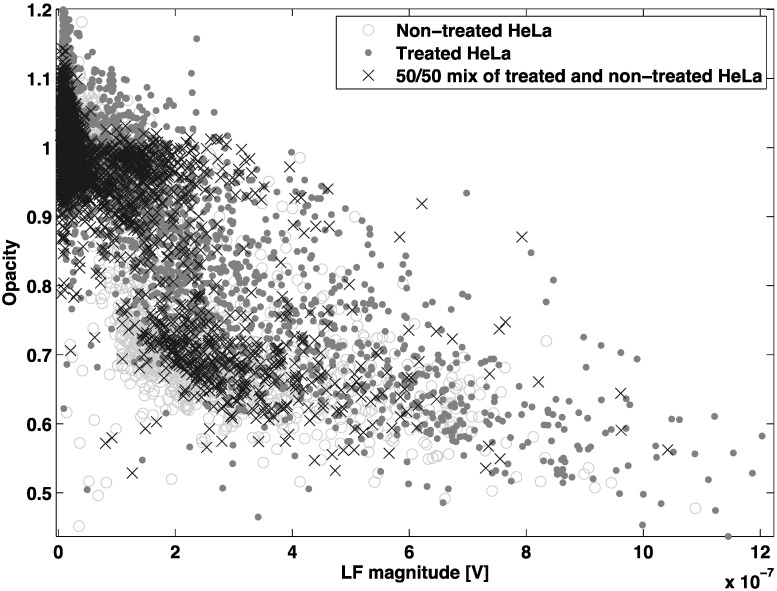
Scatter plot of amplitudes for LF = 82 kHz and HF = 1.57 MHz. The figure shows the opacity response as a function of the LF signal of three different sample mixtures; beads and treated cells, beads and non-treated cells and bead plus treated and non-treated cells. The 4.5-µm beads from the three sets are visible in the top left corner centered on one.

**Figure A3:**
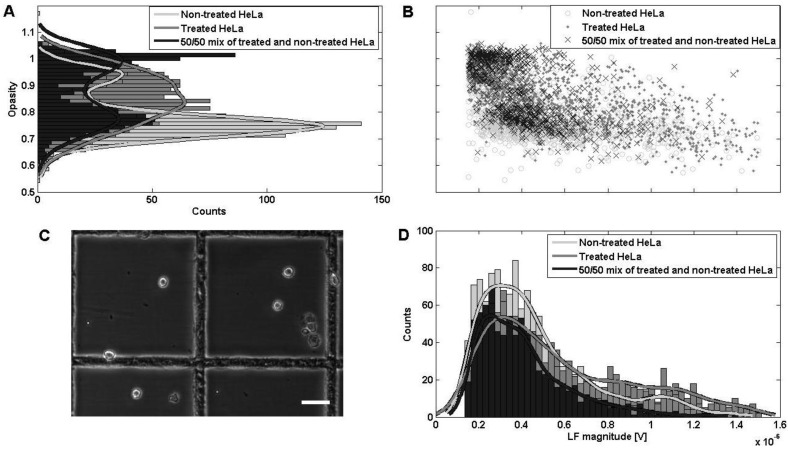
Data analysis of the impedance response of treated and non-treated cells; the frequencies used were high frequency (HF) at 1.57 MHz and low frequency (LF) at 210 kHz. (**A**) Histogram distribution of the opacity (HF/LF) of the different samples; beads and treated cells, beads and non-treated cells and bead plus treated and non-treated cells. (**B**) Amplitude scatter plot of the opacity response as a function of the LF signal of three different samples. (**C**) Optical image of the used cell sample with a 50/50 mix of treated and non-treated cells stained with trypan blue (scale bar: 50 µm). (**D**) Histogram distribution of the LF signal.

Three samples of cells were prepared: one with non-treated HeLa cells, one with treated HeLa cells and one mix sample of treated and non-treated cells. In all samples, 4.5-µm beads were added in the same concentration as the cells at 0.5 × 10^6^ particles/mL. Figure 5A shows the maximum differential impedance scatter plot for the impedance characterization of the cells treated with paclitaxel and non-treated cells. Figure 5B,C represents histograms of the opacity and LF voltage, respectively. The frequencies used in Figure 5 are LF = 82 kHz and HF = 1.57 MHz. Similar results were obtained using LF = 210 kHz (see Appendix Figure A3). The 4.5-µm beads were added to the sample in order to identify the different populations from cell debris, as cell debris is considered a waste product and not interesting for this analysis. Further, as the channel system was closed with a soft lid of PDMS, the dimensions could vary, thus changing the detected volume. This could influence the measurements, making the bead calibration a way to validate the measurement. In this way, the impedance from the beads was used as a calibration standard. For clarification, the bead responses were removed from the final data analysis (for further elaboration, see Figure A2). The opacity of the treated cells gave an average value close to that of the beads, around one, while the non-treated cells have a lower average opacity, clearly making two distributions. The optical image in Figure 6 shows both alive (red arrow) and dead cells (green arrow). A manual cell count of the stained cells verified the sample composition of approximately 50% alive and 50% dead cells in the mixed sample. The samples of the treated cells and non-treated cells were also stained with trypan blue, and a manual count of the cells showed good agreement with the reported effect of paclitaxel.

The opacity of the mix sample showed two well-defined populations representing the treated and non-treated cells. The opacity of the non-treated cells was in good agreement with the mixed sample. However, the opacity of the treated sample seems to be more widely spread. This may be caused by, e.g., drug efficiency, sample handling and channel geometry. A Student’s *t*-test was performed on the opacity data sets and gave a *p*-value of less than 0.0001. This indicates that the reported system can detect the effect of paclitaxel on cells compared to the non-treated cells. Furthermore, our system could detect a larger size distribution of the non-treated cells, indicated by the longer tail in Figure 5C.

The theoretical MMT model illustrated in Figure 3 shows that the opacity response of a non-treated cell should be around 0.6 and one for treated cells for the frequencies used. The measured opacity for the cell populations shows good agreement with the theoretical opacity and, hence, the model; the model predicts the observed trend in the measured opacity. The theoretical model is a quantitative representation of the EIS system and does not give absolute values, as the values of *σ_mem_* and ε*_mem_* are based on qualified guesses. Further, the MMT model does not take into account the stray capacitance. Thus, it still predicts the impedance response of the sample based on our knowledge of how paclitaxel affects cells.

## 4. Conclusions

In conclusion, we have fabricated and characterized a microfluidic biosensor system capable of probing the viability of samples in continuous flow with electrical impedance spectroscopy. In this study, particle diameters of 4.5 to approximately 15 µm were investigated. We have used this biosensor to characterize HeLa cells treated with paclitaxel and differentiated them from non-treated cells. The biosensor was able to distinguish between the two types of cells, shown by two populations in the opacity (with *p* < 0.0001). The opacity is a parameter known to reflect changes in the cell membrane. In accordance with the Maxwell mixture theory, the impedance response of the opacity for the treated cells increased compared to non-treated ones. This proof of concept report shows that EIS can be used as a tool for analyzing the chemotherapeutic drug effect on cell membrane morphology. Thus, the method has opportunities in different therapeutic research fields, as it has the potential to extract information on the status of a cell population in ways optical methods cannot.

## Appendix

### Electrical Impedance Spectroscopy: Viability Study of Paclitaxel Treated HeLa Cells in Continuous Flow

#### Frequency Selection

The selection of the frequencies used in this work is based on frequency sweeps of the electrodes (see Figure A1). The sweeps were performed using an HF2IS Impedance Spectroscope (Zurich Instruments, Switzerland) together with the HF2TA current amplifier (Zurich Instruments, Switzerland), as illustrated in Figure 1. This was done in order to characterize the electrodes and any influence from the current amplifier on the frequency sweep. The sweeps were made from 10 kHz to 20 MHz with a time constant of 3 ms, a two-point averaging and a value of the trans-impedance of the amplifier of 1 kΩ.

Figure A1(A) shows the magnitude of the impedance. In the plot, the 80 kHz to 2 MHz response approximately represents the impedance of the channel [19]. We chose frequencies (82 kHz, 210 kHz, and 1.57 MHz) in this range for our experiments in order to obtain the best signal from the channel.

#### Data Normalization Using Beads

Beads were mixed with the cell samples to function as a calibrating standard in order to compare data from different experiments. The beads were 4.5 µm in diameter and were chosen, as they gave a lower LF response compared to the HeLa cells, which have an average diameter of 15 µm. Different data sets were then compared by aligning them to each other based on the positions of the beads. The opacity of the beads was normalized to one for all experiments. The data points from the beads were removed from the data sets before further analysis (see Figure 5). This was done by setting a minimum threshold value for the LF signal equal to 0.9 × 10^−7^ V for Figure 5. The minimum LF threshold value was chosen based on the bead experiments. Figure A2 shows the data without applying this threshold.

Figure A3(A,B) shows two distinguishable populations representing the treated and non-treated cells. The same trend is visible in Figure 5.

#### Theoretical Model

The theoretical model uses a three-step transformation of the electrical field, as described by [23,26], which transforms the field generated by the coplanar electrodes into a parallel electrode configuration with a linear field. This gives an electrical field in the transformed space as *E_trans_ = V/2K(k^2^)*, where *E_trans_* is the field in the transformation, *V* the potential between the electrodes and *K(k^2^)* is the complete elliptic integral of the first kind with modulus *k^2^* (see [23]). The cell constant is given by *κ* = 2*K(k^2^)*/*K(1 − k^2^)*. The impedance responses of a cell (*z_mixc_*) and a bead (*z_mixb_*) are calculated based on the Maxwell mixture theory [18], which gives values for the different parts as:

(1)

(2)

(3)

(4)

(5)

(6)

(7)

(8)

(9)

(10)

The total impedance of the system with a cell between one set of electrodes is:

(11)

The total impedance of the system with a bead between one set of electrodes is:

(12)

where  is the empty channel. The calculations were done with the following parameters: *j*^2^ *=* −1, *l =* 30 µm, *w =* 30 µm, *ε*_0_ *=* 8.854e−12 F·m^−1^, *r_bead_* *=* 2.5 µm, *σ_bead_* *=* 1e−3 S·m^−1^, *ε_bead_* *=* 2.6*ε*_0_, *r_cell_* *=* 7.5 µm, *d_cell_* *=* 5 nm, *σ_mem_* *=* 10e−8 S·m^−1^, *µ_mem_* 8.3*µ*_0_, *σ_m_* *=* 1.6 S·m^−1^, *µ_bead_* *=* 80*µ*_0_, *σ_i_* *=* 0.6 S·m^−1^, *µ_i_* *=* 60*µ*_0_, *k* *=* 1.3713; where the frequency *f* is given as *f* *=* 2*πω* and was varied from 60 kHz to 10 kHz.
